# Young Academician Network (YAN) Project: Creating a Sustainable Ecosystem of Training for Early-Career Healthcare Student Researchers

**DOI:** 10.1192/bjo.2022.152

**Published:** 2022-06-20

**Authors:** Jiann Lin Loo, Jashan Selvakumar, Sahar Ali, May Honey Ohn

**Affiliations:** 1 Betsi Cadwaladr University Health Board, Wrexham, United Kingdom; 2 St George's University of London, London, United Kingdom; 3 Keele University School of Medicine, Keele, United Kingdom; 4 Croydon University Hospital, London, United Kingdom

## Abstract

**Aims:**

A lot of healthcare students are interested to have early involvement in research and one of the common obstacles is getting access to a mentor who can help them venture into academic work. Therefore, the Young Academician Network (YAN) project has been conceptualised in November 2020 after an opportunistic email communication between a medical student and a psychiatrist registrar, with the vision of creating a sustainable ecosystem of mentoring in research. This article is aimed to elucidate the journey of the YAN project and the lessons learned after a year.

**Methods:**

The word YAN originates from the Mandarin word for “research”, which is the theme for the project. The mission is to train healthcare student research leaders who will be able to lead their juniors into the field of research. It began with a weekly hourly online meeting between the student and registrar with the agenda of brainstorming research ideas, reflections from the previous meeting, reviewing the progress of tasks, and discussions of topics that were relevant to research. All explored research topics were discussed based on SMART (specific, measurable, achievable, relevant and time-bound) goals to ensure they were feasible since there was no external funding involved.

**Results:**

The YAN project had successfully published one full article in a peer-reviewed journal and two proceedings in an international congress within a year. Meanwhile, there are two ongoing projects with abstracts produced for submission to different international conferences. The lack and restriction of resources led to the promotion of creativity rather than stunted growth of the project. The main challenge of the project was the difficulty in meeting the dateline due to the busy timetable of different members. Other challenges included the difficulty of striking a balance between vision and reality.

**Conclusion:**

As this is a not-for-profit initiative, a high level of motivation is required to keep the project moving forward. Although the number of participants has not grown significantly, this pilot project has at least shown its feasibility without any funding support. There is a plan for further expansion of the project to recruit more members once the foundation of this project has been established with an adequate number of publications. A more structured and systematic evaluation of this project is needed to provide vital information for further improvement of this project.

